# First Annual Report of the Supervising Surgeon of the Marine Hospital Service of the United States

**Published:** 1873-04

**Authors:** 


*First Annual Report of the Supervising Surgeon of the Marine Hospital Service of the United States*, for the year 1872, containing a brief Historical Sketch of the Service from its Organization in 1798. Washington: Government Printing Office.

This volume consists of about one hundred pages, and embraces an interesting History of the service from its first organization in 1798 to the present date. The law of 1798 required American seamen to pay a tax of twenty cents per month to the various collectors of customs. Out of this fund the President was authorized to provide for the temporary relief of sick and disabled seamen, the surplus was to be devoted to the erection of Marine Hospitals. This law was also made on the year following to embrace officers and seamen of the Navy. By an act of 1870 the tax was increased to forty cents per month to be collected from each seaman employed on American vessels. Fishing vessels and canal boats were not included. This act also provided for the appointment of a supervising surgeon of the U. S. Marine Hospital Service. This office was filled in April, 1871, by the appointment of John M.

Woodworth, M. D., whose thorough acquaintance with his profession and the duties of his office makes him eminently fitted for the place. As an evidence of the watchful care exercised by this officer, we mention the following item. For the year ending June 30, 1871, the everage expense per patient was $1.04 per day against an average of 97.6 cents per day for 1872, notwithstanding the fact that many of the seamen were afflicted with small-pox, which would tend to increase the expense. The Surgical Department of the work comprises a brief report of over four hundred cases. This is followed by several statistical tables. The report is very well made and is worthy of much commendation.

